# Risk factors and predisposing conditions for amniotic fluid embolism: a comprehensive review

**DOI:** 10.25122/jml-2024-0406

**Published:** 2025-04

**Authors:** Jumana Hussain Timraz, Ruqayyah Ali Ahmed, Nada Yasser Metwali, Zenab Javed, Shahd Abdelazim, Raabeah Farhan, Faten Ahmad Yaseen, Hossam Abdelfatah Mansour

**Affiliations:** 1 Department of Medicine and Surgery, Batterjee Medical College for Science and Technology, Jeddah, Saudi Arabia; 2 Department of Obstetrics and Gynecology, Aya Specialist Hospital, Jeddah, Saudi Arabia; 3 Department of Obstetrics and Gynecology, Mansoura University Hospital MUH, Mansoura, Egypt

**Keywords:** amniotic fluid embolism, coagulopathy, postpartum, fetal debris

## Abstract

Amniotic fluid embolism (AFE) is a rare, yet life-threatening obstetric emergency characterised by sudden collapse of the mother due to circulatory and respiratory failure, often accompanied by coagulopathy. It accounts for a significant proportion of peripartum cardiac arrests and maternal deaths, with an incidence of 2–8 per 100,000 deliveries. The specific pathophysiology behind AFE remains unclear. However, one hypothesis states that amniotic fluid or fetal debris enters the maternal circulation, triggering a severe inflammatory and immunologic response. Diagnosis of AFE is primarily clinical as it relies on exclusion due to the unavailability of any definitive diagnostic test. Risk factors include caesarean delivery, multiple pregnancies, advanced maternal age, and pre-existing health conditions or comorbidities. Effective management centres on early recognition, aggressive, urgent supportive measures, and resuscitation. Advanced therapeutic options, such as veno-arterial extracorporeal membrane oxygenation (VA-ECMO), have shown potential in severe cases. Despite medical advancements in supportive care, which have led to reduced mortality rates, AFE remains highly unpredictable, carrying a significant risk of maternal and fetal mortality and morbidity. Survivors are often faced with long-term complications such as neurological deficits and cardiac problems. This comprehensive review aims to improve clinicians’ awareness of AFE, summarize its risk factors, and provide an overview of the current strategies for early recognition and management, emphasizing recent advancements and the need for continued research in this critical area.

## INTRODUCTION

Amniotic fluid embolism (AFE) is an uncommon but often catastrophic obstetric emergency, characterized by abrupt maternal collapse due to severe respiratory and circulatory failure accompanied by coagulopathy. It most frequently occurs during labor or the immediate postpartum period and represents the second leading cause of maternal death in the United States, as well as the primary cause of peripartum cardiac arrest [1]. The first suspected case of AFE dates back to Brazil in 1926, but it was Steiner and Luschbaugh who, in 1941, provided the initial pathological description by identifying fetal cells in the pulmonary circulation of women who died during labor. Global incidence estimates vary widely, from 1 in 8,000 to 1 in 80,000 deliveries, largely because standardized diagnostic criteria and reporting protocols remain lacking [2]. Most recent data suggest a prevalence of 2–8 cases per 100,000 pregnancies [3].

AFE typically presents with a clinical tetrad of sudden hypotension or cardiopulmonary arrest, disseminated intravascular coagulation (DIC), absence of fever, and onset during labor or the immediate postpartum period [4,5]. In the absence of any specific diagnostic test, early recognition of these four features is critical. Although the precise etiology of AFE remains unknown, current evidence implicates the entry of amniotic fluid or fetal debris into the maternal circulation as the triggering event [6]. AFE is one of the leading causes of unexpected obstetrical death. Recently, it has been often referred to as an anaphylactic syndrome of pregnancy involving the activation of the coagulation cascade, causing vasospasm, edema, and early onset of DIC, which is one of the potential causes of sudden death in obstetrics. The symptoms are variable and may precede, occur during, or follow delivery. Patients classically presenting with sudden changes in mental status and cardiac arrest, or severe hypotension characterized by the presence of DIC, may be a vital presenting feature. Recognized risk factors include maternal age over 35 years, cesarean delivery, multiple gestations, operative vaginal delivery (forceps or vacuum), amniocentesis, induction of labor, polyhydramnios, placental abruption or previa, uterine rupture, and abdominal trauma [4].

Because no definitive test exists for amniotic fluid embolism, the diagnosis remains one of exclusion and hinges on rapid clinical evaluation. Early recognition is crucial, and immediate supportive measures, such as airway stabilization, aggressive fluid resuscitation, hemodynamic support, and correction of coagulopathy, can dramatically improve maternal and fetal survival [4]. Therefore, early recognition and effective treatment are more significant than prompt diagnosis. This necessity for exclusion also undermines research: without clear diagnostic criteria, it remains difficult to establish accurate incidence rates, identify true risk factors, evaluate management strategies, or track outcomes. The management for AFE is primarily supportive. Rapid recognition, effective resuscitation, and anticipation of coagulopathy and severe postpartum hemorrhage are crucial in promoting patient survival. Recent innovations such as veno-arterial extracorporeal membrane oxygenation (VA-ECMO) emphasize the need for a multidisciplinary approach. The clinical introduction of ECMO with an antithrombotic circuit provides new therapeutic options [1]. Recently, VA-ECMO has been effectively used to manage patients with minimal complications. It was achieved by controlling bleeding through massive transfusion, utilizing an antithrombotic ECMO circuit, and postponing the start of anticoagulant and anti-DIC medications [5].

This comprehensive review aimed to (1) raise awareness of AFE as a potential etiology in any unexplained antepartum or postpartum collapse; (2) synthesize the historical evolution, epidemiology, and proposed pathophysiology of AFE; (3) critically appraise the clinical presentation and established risk factors that inform early recognition; (4) evaluate current diagnostic challenges and exclusionary approaches in the absence of definitive tests; (5) review evidence-based management strategies—including standard supportive measures and emerging therapies such as VA-ECMO; and (6) identify key gaps in knowledge and propose targeted directions for future research to improve maternal and fetal outcomes.

### Epidemiology and prevalence

Amniotic fluid embolism occurs in approximately 2–8 per 100,000 deliveries worldwide and is responsible for 5–15% of direct maternal deaths, with case fatality rates ranging from 11% to 44% [7]. Although incidence estimates vary, 2–8 per 100,000 in the United States and United Kingdom, and comparably high rates in Australia, AFE accounts for 5–15% of maternal deaths globally. With eight out of twelve cases in 2011, AFE was the most common cause of death directly related to childbirth in Germany; however, only one case had an autopsy. Several factors modify AFE risk. Cesarean delivery carries an incidence of 22 per 100,000, nearly three times that of vaginal birth (8 per 100,000). Maternal age also influences rates: women aged 30–39 face an incidence of 17 per 100,000 compared with 8 per 100,000 among those aged 15–29. Despite stable incidence over recent decades, improvements in critical care have driven a significant decline in AFE-related mortality in developed settings [7].

### Pathophysiology and underlying mechanism

**Figure 1:**
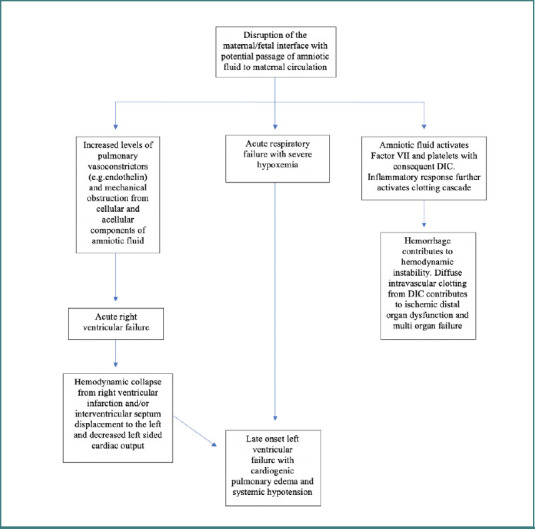
Pathophysiology of AFE

Three distinct pathophysiologic mechanisms are thought to be involved in amniotic fluid embolism syndrome cases. These three contributing pathways are: (a) mechanical blockage of vessels brought on by amniotic fluid embolism, (b) inflammatory response that amniotic fluid embolism subsequently causes in the mother's circulatory system, and (c) an immunologic mechanism that is still not fully understood [7]. When uterine trauma causes amniotic fluid to physically breach the barrier separating the mother and fetal environments, the clinical syndrome itself is triggered [8]. The pulmonary vasculature of the mother contains fat from the vernix or infantile mucin, lanugo hair, and epithelial squamous cells, according to autopsy reports from fatal cases of AFE [8]. The molecular and cellular mechanisms underlying AFE involve several key pathways. AFE is triggered by the entry of amniotic fluid into the maternal circulation, leading to systemic inflammation, coagulopathy, and vascular collapse. Amniotic fluid components activate immune cells and release pro-inflammatory cytokines, causing endothelial dysfunction and pulmonary edema. Tissue factor from the fluid initiates the coagulation cascade, resulting in disseminated intravascular coagulation [7,8]. Additionally, vasoactive substances like endothelin induce pulmonary vasoconstriction, increasing vascular resistance and leading to right ventricular failure [8]. These molecular and cellular pathways collectively explain the rapid and severe clinical manifestations of AFE. Moreover, the lower uterine segment and small endocervix tears, commonly observed during labor and delivery, are suspected sites of amniotic fluid entry into the mother's circulation. Once amniotic fluid enters the mother's systemic circulation, it can block various circulations, including the pulmonary circulation [8]. A proposed flowchart of the pathophysiology of AFE is shown in Figure 1.

### Risk factors and predisposing conditions

Although AFE is rare and unpredictable, some factors can heighten its probability of occurrence. These have been grouped into four broad categories: maternal characteristics, obstetric factors, procedural factors, and miscellaneous conditions.

### Maternal characteristics

Advanced maternal age has consistently been identified as a risk factor for AFE. It was recorded that women ≥ 35 years experience higher AFE incidence rates and fatality rates [9]. Other factors, including multiparity, multiple gestation, male fetuses, previous caesarean sections, Asian and black races, and early gestational age, contribute to the development of AFE. Additionally, women with conditions such as gestational diabetes, cardiac disease, cerebrovascular disorders, and renal disease have a higher risk [9,10].

### Obstetric factors

Most AFE risk factors are linked to circumstances that increase maternal-fetal interface disruption, encompassing placental abnormalities, specific delivery methods, and other conditions [11].

### Placental abnormalities

Some abnormalities, such as placenta previa, placental abruption, and placenta accreta, are thought to be associated with AFE. Recent studies have suggested that the placenta accreta spectrum (PAS) may be involved in the pathogenesis of AFE, which may facilitate the entrance of shaped amniotic fluid components into the mother’s circulation [11]. Subsequently, exposure to fetal or trophoblast substances may result in the production of inflammatory mediators in pregnant individuals, causing severe acute reactions and eventually developing AFE. Placental abruption during late-preterm or early-term gestation in individuals with PAS can lead to a high incidence rate of AFE. However, this is an infrequent clinical condition [11-13]. According to a case report that was published in 2022 of a pregnant female with placental mesenchymal dysplasia (PMD) complicated by AFE and intrauterine fetal demise (IUFD), it was notable that PMD could result in cumulative risk factors contributing to AFE, including placenta previa totalis, in addition to the increased extent of disruption of the maternal-fetal interface that may occur during manual expulsion of the placenta because of the wide attachment of the placenta to the endometrium. However, the link between PMD and the likelihood of AFE is not fully understood because it was the first reported case of PMD complicated with IUFD and AFE [14,15].

### Delivery methods

It is noteworthy that there is a strong association between the way of delivery and AFE occurrence rates. Both cesarean section, and instrumental vaginal delivery with suction cup or forceps are considered factors that raise the chances of developing AFE [13,14]. Also, induction of labor is considered a risk factor [16,17].

### Other conditions

Cases with particular pathological conditions are found to have a higher risk of AFE, including cervical laceration and abdominal trauma. In addition, the risk of AFE is increased in cases of uterine rupture because of the damage inflicted upon the uterine myometrium and venous sinuses [18,19].

### Miscellaneous risk factors of AFE

#### Eclampsia/Pre-eclampsia

Pre-eclamptic women typically maintain their blood pressure during regional blockade; routine fluid loading is superfluous and may exacerbate the condition of fluid overload. Small doses of vasoconstrictors can be used if hypotension develops. More than 500 ml of fluid replacement is typically not required, even at CS, unless it is matched against blood loss. Avoiding general anaesthesia is advised, if at all possible, as the mother's risk will increase during intubation and extubation due to increases in systolic and diastolic blood pressure and heart rate. Labetalol, alfentanil, or remifentanil can be used to lessen this [20,15].

#### Polyhydramnios

Polyhydramnios, characterized by excessive amniotic fluid volume, predisposes to several obstetric complications due to uterine overdistension. These include atypical fetal presentation, umbilical cord prolapse, and fetal macrosomia, often associated with maternal diabetes mellitus, as well as an increased risk of urinary tract infections. A prospective longitudinal study of uncomplicated singleton pregnancies reported higher rates of cesarean delivery for fetal indications and increased neonatal intensive care unit admissions among those with polyhydramnios. A large study of 85,000 pregnancies revealed that 3900 had an elevated AFI. Polyhydramnios was found to be an independent risk factor for perinatal mortality. The prognosis was the worst for a small gestational age (SGA) fetus with polyhydramnios [16].

#### Placental abruption

Perinatal death rates range widely from 2 to 67% according to reported data. Numerous known risk factors include trauma, thrombophilia, smoking, and high blood pressure. Repeated first-trimester loss as well as second- or third-trimester loss are also linked to thrombophilia. However, it is unknown if a history of these losses raises the risk of abruption. In this retrospective case-control study, 2371 control and 161 affected women were enrolled to determine risk factors for placental abruption [14]. Clinical examination of the placenta, painful contractions, and heavy vaginal bleeding beginning in the third trimester were typically used to make the diagnosis. The 13 and 26-week gestational ages were used to define trimesters. The prevalence rates of smoking, intrauterine growth retardation, pre-eclampsia, low Apgar scores, and cesarean deliveries were significantly higher in women who experienced placental abruption [19]. Compared to control women, affected women had lower first-trimester hemoglobin values; twice as many had final hemoglobin levels of less than 11.0 mg/dL. An increased risk of placental abruption was significantly correlated with prior second-trimester and repeat fetal loss. Three times as many women with pre-eclampsia were at risk. The affected women were significantly more likely to have a family history of thromboembolism (19.6% vs. 6.7%), even though none had a history of venous thromboembolism (0.4% vs. 0.4%). Risk scores were generated from the data. The risk increased from 2.5-fold for those with a score of one to nearly 100-fold in those having a score of 4 or above. The corresponding odds ratios for placental abruption were 2.7 and 94.5. Second- and third-trimester fetal losses and repeated losses were significant risk factors for placental abruption in this retrospective study. The risk score may prove helpful in deciding on appropriate monitoring in women with third-trimester bleeding or premature contractions [14].

#### Uterine inversion

Uterine inversion during the acute puerperium is an uncommon but potentially fatal obstetric emergency. If bleeding is not promptly identified and treated appropriately, it could be severe. Inversion is classified by the extent of fundal prolapse: first-degree (partial) inversion involves the fundus remaining within the endometrial cavity; second-degree inversion extends the fundus through the cervical os; third-degree inversion brings the fundus to the level of the vaginal introitus; and fourth-degree inversion represents complete eversion of the uterus and vagina through the introitus, often accompanied by progressive prolapse of the fundus [17].

In older studies, the rate of maternal mortality due to uterine inversion can reach 15%, but more recent reports indicate that this rate is much lower, especially in developed countries. Several risk factors have been identified, such as severe fundal pressure, excessive umbilical cord traction in the third stage of labour, and fundal placental implantation [19].

#### Intrauterine fetal death

In obstetrics, intrauterine fetal death (IUFD) remains a major concern, occurring in approximately 1% of pregnancies. Investigation of pregnancies complicated by intrauterine fetal death, including fetal and maternal parameters, can be helpful, as previous studies have shown [18]. For potential prevention and intervention, identifying the underlying causes of pregnancies complicated by intrauterine fetal death may be important. Nevertheless, the cause of at least 25% of stillbirths is still unknown despite extensive research [15,13].

### Procedural Factors

#### Amniocentesis

Amniocentesis involves the ultrasound-guided removal of amniotic fluid, typically after 15 weeks’ gestation, to harvest fetal cells for chromosomal analysis. Although the procedure can cause discomfort, it is generally well tolerated and does not require anesthesia. Today, approximately 5–10% of pregnant women elect to undergo amniocentesis.

Since its introduction in the 1960s, amniocentesis was initially reserved for those at highest risk of chromosomal abnormalities detectable by conventional karyotyping [21]. Early indications included advanced maternal age (>35 years), a prior history of amniotic fluid embolism (AFE), a family history of genetic disorders, or positive serum screening for trisomy 18 or trisomy 21 (Down syndrome). This practice continued until 2007. Currently, the most common indication for amniocentesis is maternal age greater than 35.

Women who had ultrasound-detected fetal structural irregularities that might be indicators of chromosomal abnormalities were also offered amniocentesis [14,22]. Before and after the procedure, it is crucial to counsel the patient about the risks and limitations of the test. Certain women who, for example, would never decide to end their pregnancy might believe that an invasive test is not necessary, especially if they do not think there is any benefit to being aware of a genetic disease before the baby is born. Even though they would not want to end the pregnancy, other women might want to go ahead and have an amniocentesis to get ready for caring for an affected child. During amniocentesis, the mother and fetus have a very low risk of injury. The main risk is the possibility of a miscarriage following the procedure; however, this risk is hard to estimate because there are not enough studies with sufficient controls, and most women who choose to undergo amniocentesis already have a higher risk of miscarriage [23].

#### Amnio-reduction

There is a higher chance of complications for the fetus and the mother in pregnancies complicated by hydramnios. It has been proposed that abnormalities related to amniotic pressure could be the mediating factor for the complications in polyhydramnios because of the elevated amniotic pressure in this condition. Hence, the goal of amnioreduction is to drain a significant volume of amniotic fluid to restore normal amniotic pressure. This will lessen maternal discomfort, enhance uteroplacental perfusion, and prolong pregnancy by lowering the risk of premature labour and membrane rupture. Regardless of the technique used, there is a chance of complications such as spontaneous premature rupture of membranes, placental abruption, or chorioamnionitis following the procedure [11].

### Clinical presentation and diagnosis

The clinical manifestations that indicate AFE are variable and usually occur near the time of delivery. AFE typically presents with sudden hypoxia and hypotension, frequently complicated by hemorrhage and noncardiogenic pulmonary edema. The majority of patients experience respiratory symptoms, including dyspnea, hypoxemia, acute respiratory distress, and subsequent respiratory failure may occur [24,25]. Also, the sudden onset of cardiovascular collapse and hemodynamic instability is common among AFE patients [26,27]. Most individuals experience cardiac arrest that arises from ventricular fibrillation or pulseless ventricular tachycardia, although it can also stem from asystole and/or bradyarrhythmia [25]. Disseminated intravascular coagulopathy during delivery has been reported as one of the main clinical features of the most severe forms of AFE [28]. There are other associated manifestations such as cough, cyanosis, and wheezing. Sudden loss of consciousness is considered the most frequent presentation of AFE. Additionally, it was reported that one-third of patients may have chills, agitation, a sudden feeling of anxiety, nausea, vomiting, and mental status alteration before the typical AFE presentation. Stroke and seizures are less common manifestations that can occur [25,26]. Individuals who present with cardiorespiratory arrest are known to have increased risks of neurologic sequelae and death [24].

AFE should be differentiated from conditions like pulmonary embolism (PE), anaphylaxis, eclampsia, and septic shock. AFE presents with sudden cardiovascular collapse, respiratory distress, and coagulopathy, typically during labor or postpartum. PE may cause similar symptoms but lacks AFE’s profound coagulopathy, and imaging such as CT angiography can confirm PE. Anaphylaxis involves acute hypotension and respiratory distress, often with urticaria and a history of allergen exposure, responding well to epinephrine, unlike AFE. Eclampsia is characterized by seizures, hypertension, and proteinuria, often preceded by preeclampsia signs. Septic shock develops more gradually, with fever and an identifiable infectious source. Unlike other conditions, coagulopathy in AFE involves disseminated intravascular coagulation with low fibrinogen levels. While imaging can rule out PE or infection, AFE remains a clinical diagnosis requiring rapid supportive care. Currently, the diagnosis of amniotic fluid embolism is mainly based on clinical observation and exclusion of other peripartum complications; early recognition and prompt management have a crucial role in prognosis. The hallmark of its diagnosis is the typical triad of coagulopathy, hypotension, and abrupt hypoxia. The manifestations mentioned earlier are also used to reach the diagnosis [29,30]. Possible differential diagnoses to rule out are acute myocardial infarction, thrombotic embolus, peripartum cardiomyopathy, septic shock, placental abruption, anaphylaxis, local anesthetic toxicity, and transfusion reaction [30]. Moreover, transthoracic echocardiography (TTE) and transesophageal echocardiography (TEE) have been noticed to be useful in excluding other alternative diagnoses, such as hypovolemia and pulmonary embolism [26].

### Management

**Figure 2:**
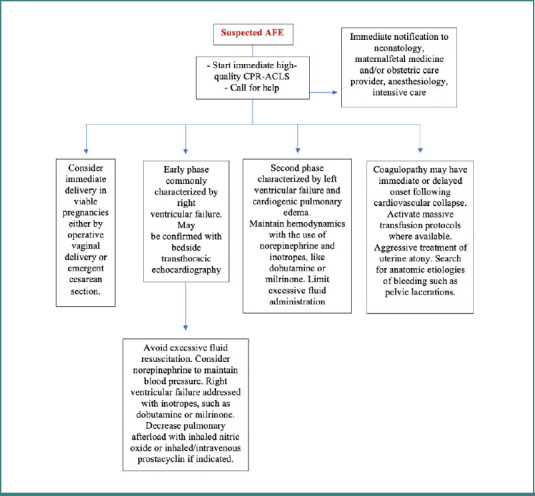
Algorithm of immediate supportive treatment in suspected cases of amniotic fluid embolism [32]

Managing amniotic fluid embolism during pregnancy is challenging due to the nonspecific hemodynamic features inherent to pregnancy, the variability of the clinical manifestations, and the diversity of risk factors [25]. It necessitates advanced supportive techniques and a multidisciplinary team involving members from anesthesia, surgery, critical care, cardiology, maternal-fetal medicine, hematology, and respiratory medicine [24,26]. Prioritizing immediate delivery is a major step in management to ensure the improvement of the newborn’s condition [31]. For better outcomes for both the mother and the fetus, clinicians must have a high degree of suspicion in the presence of suggestive symptoms, along with a quick response to clinical signs of severe fetal distress or cardiopulmonary arrest [25]. Cardiopulmonary stabilization with resuscitation efforts and treatment of hypoxia is one of the main strategies to maintain cardiopulmonary arrest. Additionally, ECMO therapy is also useful; involvement of the ECMO team is essential for providing cardiopulmonary support. Maintenance of vascular perfusion and correction of coagulopathy are crucial in managing AFE. In addition, the use of transesophageal echocardiography (TEE) and transthoracic echocardiography (TTE) is beneficial in guiding intraoperative clinical management [26,29]. Anesthetic interventions that target the altered vital signs early might delay the onset of some symptoms in individuals experiencing AFE during caesarean birth. The long-term complications that may develop, including acute kidney failure, neurological sequels, non-cardiogenic pulmonary edema, recurrent arrhythmias, and cardiac ischemia, need monitoring and individualized management. Furthermore, it is extremely important to promptly address the neurological damage that results from prolonged in utero hypoxia in newborns, and these patients must be included in the long-term follow-up by trained personnel [25]. Figure 2 demonstrates an algorithm applied in the immediate management of suspected cases of AFE.

### Prognosis & outcomes

Amniotic fluid embolism is an uncommon but life-threatening obstetric emergency characterized by high maternal and perinatal morbidity and mortality. Early reports placed the maternal mortality rate as high as 61%, but more recent data suggest it is closer to 10%. Of those affected, roughly half die within the first hour of onset, and two-thirds succumb within 5 hours; the highest risk period is between one and 12 hours after AFE begins. The risk of recurrence is uncertain, but examples of successful subsequent pregnancies have been documented. In future pregnancies, recommendations for elective cesarean delivery to reduce labour-related risks remain controversial. Infant mortality rates are about an estimated 30%, with survivors facing increased risks of hypoxic-ischemic encephalopathy, cerebral palsy, and cognitive impairments. In addition, statistics show that stillbirth and neonatal death rates can range from 10% to 40%. However, advances in diagnosis, clinical management, and deeper understanding of AFE pathophysiology can improve survival rates of the mother and the fetus. Early recognition and intervention have benefited the maternal outcomes as well as the neonatal mortality and morbidity rates. Long-term complications, including neurological deficits, cardiac issues, or psychological effects such as depression and post-traumatic stress disorder (PTSD), could be experienced by the survivors of AFE. Mothers and infants often require intensive care and close monitoring, emphasizing the significance of immediate and effective management strategies to improve outcomes [2]. Survivors of AFE may face long-term neurological and psychological sequelae due to hypoxic injury and the traumatic nature of the event. Neurological issues include cognitive impairments such as memory deficits, attention difficulties, and, in severe cases, hypoxic-ischemic encephalopathy. Psychologically, survivors are at heightened risk for PTSD, anxiety, and depression. Management involves early neurological assessment and physical, occupational, and speech therapy rehabilitation to improve functional outcomes. Psychological support, including counseling and cognitive-behavioral therapy (CBT), can help address PTSD and emotional distress. Participation in support groups and family education further enhances coping and recovery. Regular follow-ups with a multidisciplinary team are essential to comprehensively address neurological and psychological needs [2].

### Implications

AFE remains a critical challenge due to its sudden onset and high mortality. Rapid response strategies and standardized, evidence-based protocols are essential to improve maternal and fetal outcomes, supported by specialized training for all healthcare providers involved in obstetric emergencies. Comprehensive follow-up care is necessary to address the long-term potential outcomes experienced by the survivors. Maternal rehabilitation is required as survivors may require long-term medical care to manage complications such as cardiovascular or neurological issues. Specialized training and established protocols are needed for healthcare providers to manage AFE effectively. Emergency response requirements, including intensive cardiovascular and respiratory support as well as urgent delivery of the fetus, must be available at all times. Because AFE is rare, continued research is imperative to identify risk factors, refine diagnostic methods, and develop targeted therapies. While AFE cannot always be prevented, preventive measures such as understanding risk factors and improving prenatal care might help in early identification and potentially alleviate some risks.

### Future directions

Despite advances in obstetric care, our understanding of AFE remains limited by its rarity and clinical complexity. Future research should focus on elucidating its pathophysiological mechanisms, developing sensitive and specific diagnostic tools for earlier detection, and refining therapeutic strategies to stabilize both mother and fetus and correct coagulopathy. Standardizing emergency response protocols and expanding hands-on training for obstetric teams will enhance rapid, effective management. Prospective, long-term studies of AFE survivors are also needed to characterize chronic sequelae and guide optimal post-acute care. Increasing public and professional awareness about AFE can lead to better preparedness and potentially improve outcomes through early recognition and intervention.

Educating patients about the signs of AFE and the importance of immediate medical attention can facilitate early identification and improve overall management. Developing telecommunication for remote monitoring and consultation can provide timely management and support, especially in underserved areas where specialized care might be limited. The era of artificial intelligence (AI) is just around the corner, so implementing AI and machine learning algorithms to analyze large datasets can enhance predictive models for AFE, improving diagnostic accuracy.

## CONCLUSION

Amniotic fluid embolism remains a rare but catastrophic emergency in the field of obstetrics, which requires rapid recognition and early multidisciplinary intervention in order to improve clinical outcomes. Despite the advancements in supportive care, the unpredictable nature of AFE contributes to its significant maternal and fetal morbidity and mortality. Continued research, improved diagnostic tools, and evidence-based management protocols are essential for the enhanced understanding and treatment of this complex but serious condition, ultimately reducing its impact on maternal health and improving maternal outcomes.
